# Lysosome-related organelles employ divergent mechanisms to modulate cytosolic zinc homeostasis

**DOI:** 10.1371/journal.pgen.1012199

**Published:** 2026-06-22

**Authors:** Chaoyi Xie, Yi Luo, Yawen Zheng, Bowen Liu, Shibo Song, Jie Wei, Anbing Shi, Yanling Yan

**Affiliations:** 1 Department of Biochemistry and Molecular Biology, School of Basic Medicine, Tongji Medical College and State Key Laboratory for Diagnosis and Treatment of Severe Zoonotic Infectious Disease, Huazhong University of Science and Technology, Wuhan, Hubei, China; 2 Cell Architecture Research Center, Huazhong University of Science and Technology, Wuhan, Hubei, China; UC Berkeley: University of California Berkeley, UNITED STATES OF AMERICA

## Abstract

Elevated environmental zinc levels pose significant toxicity to biological systems, necessitating adaptive responses to mitigate excessive zinc exposure. In *C. elegans*, a specific lysosome-related organelle, the gut granule, may increase in number and volume with high dietary zinc, thereby lowering cytosolic zinc concentration, though the mechanisms remain unclear. Our results suggest that GLO-1 predominantly controls granule biogenesis, whereas zinc-induced granule expansion involves distinct mechanisms. Further study revealed that high zinc upregulated GLO-1 activity through its GEF complex GLO-3-CCZ-1, by enhancing transcription of GLO-3 and post-translational modification of CCZ-1. Zinc transporter CDF-2 has been identified to mediate zinc influx into gut granules. In this study, analysis of 14 *C. elegans* CDFs reveals that ZK185.5 (CDF-3) and F19C6.5 (CDF-4) also localize in gut granules. Functional studies suggest that CDF-3, not CDF-4, complements CDF-2 in facilitating zinc influx into gut granules. Unlike CDF-2, the expression of CDF-3 is downregulated in a high zinc diet. These results suggest a modulation in the composition of CDFs within gut granules in response to environmental zinc. Together, our study reveals a sophisticated zinc detoxification mechanism of *C. elegans* gut granule to uphold cytosolic zinc homeostasis amidst fluctuating environments.

## Author summary

Maintaining zinc homeostasis is critical for the survival of free-living *C. elegans*, in which the lysosome-related organelle, the gut granule, plays a pivotal role. In this work, a divergent mechanism adopted by gut granules to protect the animal from high zinc toxicity is revealed. First, zinc stimulates GLO-1 activity through regulation of its GEF complex, which may contribute to gut granule biogenesis. Second, a new zinc transporter, CDF-3, was found to localize to gut granules and mediate zinc influx as CDF-2. Strikingly, unlike CDF-2, the expression of CDF-3 was downregulated upon high zinc treatment, indicating that gut granules rearrange their CDF composition to adapt to a high zinc diet. This study provides new insights into the zinc homeostasis mechanism in the nematode *C. elegans*.

## Introduction

Zinc is essential for various tissues, particularly in the brain, pancreas, and immune system organs. Approximately 10% of the human proteome consists of zinc-binding proteins. Notably, zinc is crucial for about 2,700 enzymes [1], facilitating catalytic processes and maintaining protein structural integrity. Through these enzymatic actions, zinc participates in numerous cellular biochemical processes [2, 1]. Moreover, proteins containing zinc finger domains serve diverse biological functions, especially through interactions with DNA, RNA, and poly-ADP-ribose [3]. Recent studies further revealed zinc’s significance as an intracellular signaling molecule [4, 5, 6]. These findings highlight zinc’s multifaceted role as a regulator of fundamental cellular processes, including gene expression, immune modulation, and neural signaling.

Elevated concentrations of cytosolic zinc can have adverse health effects, potentially contributing to conditions such as diabetes, cancer, inflammation, and neurodegenerative disorders [7]. To mitigate the harmful effects of zinc, cells sequester and store it in intracellular compartments to prevent cytosolic zinc overload [8]. In mammalian cells, zinc concentrations typically range from 200 to 300 μM [9], predominantly associated with proteins or contained within organelles. As a result, the cytoplasm maintains a minimal level of labile zinc, usually estimated in the picomolar range [9]. Upon specific stimulation, zinc is released from intracellular stores into the cytoplasm. In neuronal cells, synaptic vesicles store high concentrations of zinc, which are co-released with neurotransmitters during synaptic transmission. This release modulates neuronal activity by interacting with membrane receptors [10, 11, 12]. Similarly, in sperm cells, membranous organelles facilitate rapid zinc efflux, resulting in elevated cytoplasmic zinc levels and sperm activation [6]. Hence, although zinc is a crucial element in numerous biological processes, its cytosolic levels must be tightly regulated to avoid potential health issues.

The regulation of zinc homeostasis is primarily managed by the coordinated activities of two families of transporters: CDF/ZnT/SLC30 and ZIP/SLC39 [13, 14, 15]. They are highly conserved across species, including yeast, nematodes, fruit flies, zebrafish, plants, and mammals [8]. The CDF/ZnT/SLC30 family functions to lower cytoplasmic zinc concentrations by either transporting zinc into organelles or expelling it from the cells [16]. In contrast, the ZIP/SLC39 family facilitates the import of zinc from extracellular spaces or organelle lumens into the cytoplasm [17]. Humans have a total of ten CDF/ZnT/SLC30 proteins, which vary in subcellular localization [16, 18]. For example, ZnT1 and ZnT2 localize to the plasma membrane to facilitate zinc efflux [19, 20, 21], while other ZnT members are responsible for transporting zinc into different organelles, likely serving as a cellular reserve [8]. *C. elegans* features an expanded array of 14 CDF/ZnT/SLC30 homologs [22, 23]. Notable members include SLC-30A5, found in the endoplasmic reticulum, and SLC-30A9, which localizes to mitochondria in epidermal cells [24]. The *ttm-1* gene produces three isoforms, with TTM-1A localizing to intracellular puncta and TTM-1B targeting the apical membrane of intestinal cells [22]. CDF-1 and CDF-2 are located at the basolateral membrane and lysosome-related organelles (LROs), respectively [25, 22, 26]. However, the localization and physiological roles of nine additional CDFs remain uncharacterized (F41C6.7, F56C9.3, R02F11.3, K07G5.5, SUR-7, PDB1.1, TOC-1b, ZK185.5, F19C6.5). It’s significant to note that zinc deficiency or excess can impact the expression of zinc transporters. In *C. elegans*, elevated environmental zinc activates the transcription factor HIZR-1, which upregulates CDF transporters like CDF-2 and TTM-1B to reduce cytoplasmic zinc levels [27, 28]. Likewise, mammalian ZnT1 expression increases under high zinc conditions. Conversely, in zinc deficiency, mammals promote the expression of ZIP2, ZIP4, ZIP8, and ZIP10 to enhance zinc uptake, while in *C. elegans*, the transcription factor ELT-2 and the mediator subunit MDT-15 work together to activate ZIPs expression, facilitating zinc influx into the cytosol [29]. Together, these evolutionary conserved mechanisms ensure precise control over zinc distribution in response to varying environmental conditions.

*C. elegans*, as a free-living organism, consistently adjusts to changing environmental conditions. Its intestinal cells, being the primary interface with the external environment, are specialized in detecting and responding to nutritional fluctuations. Gut granules, a specific type of LRO [30], have evolved to meet this demand by storing essential micronutrients, including zinc and heme [25, 31]. Our research shows that the gut granule biogenesis and functionality can adapt to fluctuations in zinc levels. In high zinc environments, the expression of GLO-3/GEF and the tyrosine phosphorylation of CCZ-1/GEF are notably increased, resulting in heightened GLO-1/Rab32/Rab38 activity. This activity promotes an increase in the number and size of gut granules, thereby expanding their capacity to store zinc. It has been noted that during zinc stress, CDF-2 is upregulated and serves as the primary regulator of zinc storage in LROs [25]. Interestingly, gut granule loss *glo-1* mutants exhibit greater sensitivity to zinc compared to *cdf-2* mutants, suggesting the presence of additional zinc-buffering mechanisms within gut granules. Further exploration has identified two novel members of the CDF family, ZK185.5 (CDF-3) and F19C6.5 (CDF-4), localized in gut granules. Functional analysis reveals that CDF-3 complements CDF-2 in the facilitation of zinc transport into the granule lumen. These findings establish gut granules as intricate zinc storage sites in nematodes, utilizing organelle restructuring and transporter regulation to enhance survival in varying zinc environments. This complex adaptation mechanism likely plays a role in the ecological success of *C. elegans* in diverse habitats.

## Results

### Increased levels of dietary zinc elevate GLO-1 transcription and activation, enhancing gut granule number and capacity

**Fig 1:**
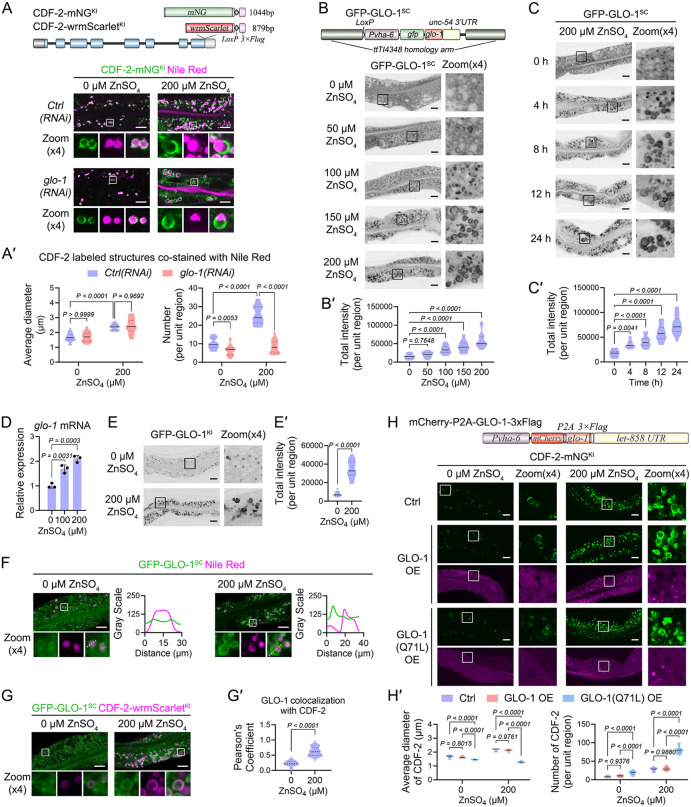
Elevated dietary zinc levels increase GLO-1 transcription and activation. (A-A’) Confocal images showing colocalization between CDF-2-mNG^KI^ and Nile Red after RNAi-mediated knockdown of *glo-1* with different zinc concentrations. Statistical analysis of the diameter and number of CDF-2-labeled structures co-stained with Nile Red was calculated manually and shown as mean ± SD (gut granule size threshold, > 0.3 μm; n = 24 regions of 3 × 3 μm^2^ from 6 animals; Two-way ANOVA with Bonferroni post-test). (**B-C’**) Confocal images showing the subcellular distribution of GFP-GLO-1^SC^ after treatment with different concentrations of zinc or at various time points of zinc exposure. An intensity threshold that exclusively encompassed vacuole-like structures in the control group under high zinc treatment was used. Statistical analysis of GFP-GLO-1^SC^ intensity was shown as mean ± SD (n = 24 circular regions of 11.9 μm diameter from 6 animals; One-way ANOVA test with Dunn’s Multiple Comparison). (D) Quantitative PCR showing *glo-1* mRNA expression levels of the Bristol N2 strain. Error bars represent 95% CIs (two-tailed unpaired Student’s *t*-test). (**E-E’**) Confocal images showing the subcellular distribution of endogenous tagged GFP-GLO-1^KI^ after treatment with different concentrations of zinc. Statistical analysis was shown as mean ± SD (n = 24 circular regions of 11.9 μm diameter from 6 animals; two-tailed Mann-Whitney test). (F) Confocal images showing colocalization between GFP-GLO-1^SC^ and Nile Red with different zinc concentrations. Line scan profiles of the two channels of the dotted yellow line were conducted using Fiji software. (G) Confocal images showing colocalization between GFP-GLO-1^SC^ and CDF-2-wrmScarlet^KI^. Pearson’s correlation coefficient was calculated using Fiji software; error bar is 95% CI (n = 18 regions of 3 × 3 μm^2^ from 6 animals). The confocal microscopy parameters in F and G: GFP-GLO-1^SC^, laser power 25%, high voltage 85; Nile Red, laser power 10%, high voltage 70; CDF-2-wrmScarlet^KI^, laser power 30%; high voltage 90; bit depth was set to 12-bit. (**H-H’**) Confocal images showing the subcellular distribution of CDF-2-mNG^KI^. Statistical analysis was shown as mean ± SD (n = 24 regions of 3 × 3 μm^2^ from 6 animals; Two-way ANOVA with Bonferroni post-test). Scale bars: 10 μm.

In high zinc diets, the volume of *C. elegans* gut granules increases, accompanied by an expansion of the membrane surface [25, 26]. Nonetheless, the mechanism driving this zinc-mediated membrane dynamics remains unidentified. Using Nile Red and endogenous CDF-2 as gut granule markers [25, 26], we did observe morphological changes in gut granules under high zinc conditions (Fig 1A). Notably, a 1.6-fold increase in gut granule number was detected following high zinc stimulation (Fig 1A’), suggesting enhanced biogenesis of gut granules in high zinc environments. Since GLO-1/Rab32 has been shown to play a pivotal role in gut granule biogenesis [30], we compared the effects of suppressing GLO-1 function under low and high zinc diets to assess the impact of GLO-1 on zinc-induced gut granule changes. A *glo-1* mutation resulted in the complete loss of gut granules, precluding observation of the effects. Thus, we utilized RNAi knockdown instead of the *glo-1* mutant. Knocking down GLO-1 in the normal diet led to a significant 35.4% decrease in gut granule number, which was further exacerbated on a high zinc diet (67.7% decrease) (Fig 1A). However, the size expansion of gut granules was not affected by GLO-1 knockdown (Fig 1A’). These findings suggest that zinc can promote gut granule biogenesis in a GLO-1-dependent manner, indicating a potential enhancement of GLO-1 activity by zinc.

The GTP-bound active form of Rab GTPase is primarily localized to membranes to exert its function, whereas the GDP-bound inactive form is predominantly distributed in the cytosol [32]. To assess the influence of zinc on GLO-1 activity, the distribution of GLO-1 was examined using confocal microscopy. To obtain low expression, a single-copy knock-in strain expressing GLO-1 under the regulation of the intestine-specific *vha-6* promoter was engineered using the CRISPR-Cas9 technique (GFP-GLO-1^SC^) [33]. Notably, GLO-1 exhibited enhanced vacuolar localization in a dose-dependent manner with zinc supplementation for 24 hours (Fig 1B). The unchanged fluorescence of the integrated *Pvha-6::gfp* strain excluded influence on *vha-6* promoter activity by high zinc treatment, indicating a specific effect on GLO-1 induced by high zinc concentration (S2A Fig). Then, the kinetic profile of zinc-dependent GLO-1 translocation was examined. Results indicated a significant promotion of vacuolar localization of GLO-1 after 4 hours of zinc exposure, with effects increasing over time (Fig 1C). In light of the upregulation of *glo-1* mRNA in Bristol N2 strain in response to a high zinc diet (Fig 1D) and the enhancement of fluorescence of endogenous tagged GLO-1 (GFP-GLO-1^KI^ strain) [34] upon zinc supplementation (Fig 1E), these findings suggest that the increased vacuolar localization of GLO-1 in the context of a high zinc diet likely arises from both transcriptional upregulation and enhanced activation. Furthermore, colocalization analysis was performed to characterize the localization shift of GLO-1. The findings revealed that zinc-stimulated GLO-1 vacuolar structures enveloped the Nile Red signal and exhibited overlap with CDF-2 (Fig 1F-G), indicating an enhanced positioning of GLO-1 on the gut granule. To further clarify the function of GLO-1 in gut granules’ adaptation to a high zinc environment, the effect of GLO-1 overactivation through dominant-active GLO-1(Q71L) overexpression in intestine (Pvha-6-mCherry-P2A-GLO-1–3 × Flag) was detected [35, 36]. When exposed to either a normal diet or a diet enriched with zinc, the overexpression of GLO-1(Q71L) resulted in a rise in gut granule number (Fig 1H). Meanwhile, we observed a decrease in the average diameter of gut granules upon GLO-1(Q71L) overexpression, which was further amplified under a high‑zinc diet. A large number of nascent organelles are small in size, which may explain this phenotype (Fig 1H’). These results are consistent with the findings in Fig 1A, with GLO-1 knockdown leading to gut granule number decrease, but not volume change. Together, these results suggest that elevated zinc levels induce GLO-1 transcription and activation, augmenting gut granule biogenesis, while zinc-induced granule expansion entails separate mechanisms.

The gut granules are labeled by both Nile Red and CDF-2; this identity might help us distinguish CDF-2 trafficking vesicles from mature gut granules. To explore the contribution of membrane trafficking to gut granule’s high zinc adaptation, we performed dual-color time-lapse imaging of CDF-2 and Nile Red (S1A Fig). The results showed that under normal culture conditions, most of the CDF-2-labeled structures were also labeled by Nile Red (S1A Fig), suggesting that they represent mature gut granules. These gut granule structures exhibited poor dynamic activity, indicating a relatively static state. Under a high zinc culture condition, however, numerous smaller CDF-2 positive structures appeared, most of which were not labeled by Nile Red (S1A Fig), suggesting that they might be trafficking vesicles. Notably, frequent events of vesicle trafficking and fusing with gut granules were observed (S1A Fig). Taken together, these data suggest that high-zinc conditions promote membrane trafficking toward intestinal granules. As Rabs played a pivotal role in membrane tethering and fusion, we sought to investigate their role in this zinc-stimulated gut granule enlargement [37, 38]. RAB-7 has been reported to localize on gut granules, and loss of RAB-7 results in a decrease in gut granule size in L4 stage worms under normal culture conditions [39]. Therefore, we choose to investigate the function of RAB-7 in high zinc-promoted volume expansion of gut granules using RNAi mediated knockdown. Under normal diet culturing, conditional knockout of RAB-7 using CRISPR-Cas9 (*ycx229*) phenocopied the *rab-7(ok511)* mutant, causing a significant size decrease of gut granules (S1B Fig). Moreover, the effect was more pronounced under a high zinc diet, disrupting the enlargement of most gut granules (S1B Fig). These results suggest a defect in zinc stimulated fusion of newly formed gut granules in RAB-7 depletion, although the detailed mechanism requires further investigation. Notably, the concurrent loss of GLO-1 and RAB-7 results in a reduction in number compared to RAB-7 knockout and a decrease in size compared to GLO-1 knockdown (S1B Fig), demonstrating an additive effect. In conclusion, our study suggests zinc stimulates gut granule biogenesis and enlargement depending on GLO-1 and RAB-7, respectively. Together, these two mechanisms remodel gut granules to provide more space for zinc storage.

### Zinc promotes GLO-1 activity by upregulating GLO-3 and enhancing tyrosine phosphorylation of CCZ-1

**Fig 2:**
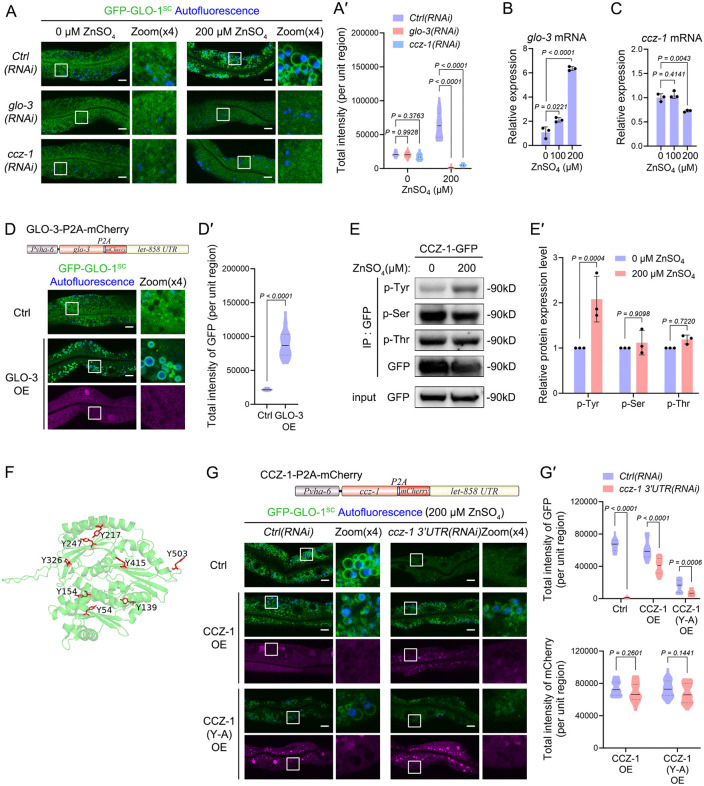
Zinc enhances GLO-1 activity through GLO-3 upregulation and CCZ-1 tyrosine phosphorylation. (A-A’) Confocal images showing subcellular distribution of GFP-GLO-1^SC^ after RNAi-mediated knockdown of *glo-3* and *ccz-1* with different zinc concentrations. Statistical analysis was shown as mean ± SD (n = 24 circular regions of 11.9 μm diameter from 6 animals; Two-way ANOVA with Bonferroni post-test). (B-C) Quantitative PCR showing *ccz-1* and *glo-3* mRNA expression levels of the Bristol N2 strain. Error bars represent 95% CIs (two-tailed unpaired Student’s *t*-test). (D-D’) Confocal images showing the subcellular distribution of GFP-GLO-1^SC^ in GLO-3 overexpression animals. Statistical analysis was shown as mean ± SD (n = 24 circular regions of 11.9 μm diameter from 6 animals; two-tailed Mann-Whitney test). The confocal microscopy parameters: autofluorescence (Ex 405 nm), laser power 20%, high voltage 110; GFP-GLO-1^SC^, laser power 25%, high voltage 85; mCherry, laser power 30%, high voltage 95; bit depth was set to 12-bit. (**E**-**E’**) Immuno-precipitation of CCZ-1-GFP and western blot analysis of its phosphorylation state. The band intensity was measured using the “Plot Lanes” function of Fiji software from three independent experiments. Phosphorylation levels were normalized to the anti-GFP signal from the IP samples and then expressed as a ratio relative to zinc-deficient conditions. The error bars represent 95% CIs (Two-way ANOVA with Bonferroni post-test). (F) Cartoon picture showing the protein structure of CCZ-1 with the potential tyrosine phosphorylation site. (**G**-**G’**) Confocal images showing the subcellular distribution of GFP-GLO-1^SC^ in different genetic backgrounds under high zinc treatment. Statistical analysis was shown as mean ± SD (n = 24 circular regions of 11.9 μm diameter from 6 animals; Two-way ANOVA with Bonferroni post-test). Scale bars: 10 μm.

Activation of GLO-1 is primarily facilitated by the guanine nucleotide exchange factor (GEF) complex GLO-3-CCZ-1 [40, 39]. We examined whether zinc-induced GLO-1 activation occurs through the regulation of GLO-3 or CCZ-1. Intriguingly, depletion of either GLO-3 or CCZ-1 through RNAi completely abolished the zinc-induced formation of vacuolar and punctate GLO-1 structures (Fig 2A). This phenotype was also evident in GLO-1 transgenic strains (S2B-C Fig), suggesting a potential involvement of the GEF factors in zinc-facilitated GLO-1 activation.

Subsequently, we measured the mRNA levels of *glo-3* and *ccz-1* across different zinc concentrations. The mRNA of *glo-3* showed a dose-dependent rise, peaking at approximately ~6-fold change with 200 μM zinc, whereas the transcription levels of *ccz-1* were minimally affected, displaying a slight decrease at 200 μM zinc (Fig 2B-C). This suggests a selective transcriptional upregulation of a GEF complex component by zinc, and its effect on GLO-1 was further detected. The ribosomal skipping peptide P2A [41] was inserted between mCherry and GLO-3 (Pvha-6-mCherry-P2A-GLO-3–3 × Flag), realizing their discrete translation and allowing the fluorescence signal to serve as a reporter of successful expression while avoiding the functional interference. To determine if upregulation of GLO-3 alone could account for zinc-induced GLO-1 activation, the GLO-3 overexpression was deployed and resulted in a significantly enhanced vacuolar localization of GFP-GLO-1 (Fig 2D). However, exerting regulatory functions through gene expression typically has a slow effect; animals may adopt quicker reactions, such as post-translational modifications. Phosphorylation is the predominant type of modification, so we aimed to determine the impact of zinc on protein phosphorylation. Minimal phosphorylation was detected in GLO-3 (S2D Fig), whereas CCZ-1 exhibited substantial Ser/Thr and Tyr phosphorylation, particularly elevated levels of p-Tyr-CCZ-1 under high zinc conditions (Fig 2E).

To assess the functional significance of CCZ-1 tyrosine phosphorylation, we utilized the Group-based Prediction System (GPS 6.0) for forecasting p-Tyr sites within CCZ-1 [42]. Then, we converted the eight potential sites to alanine residues (Fig 2F), resulting in the CCZ-1(Y-A) mutation. We utilized a *let-858* 3’UTR to enable the intestinal overexpression of CCZ-1 (wild-type and the Y-A mutation), which remains unaffected by *ccz-1* 3’UTR RNAi. Depletion of endogenous CCZ-1 via RNAi targeting of the *ccz-1 3’UTR* disrupted GLO-1 vacuolar localization, a defect rescued by wild-type CCZ-1 overexpression. However, overexpression of a CCZ-1(Y-A) leads to a dominant inhibitory effect on GFP-GlO-1 localization in the control group. Accordingly, in endogenous CCZ-1 knockdown animals, CCZ-1(Y-A) overexpression only weakly restored GLO-1 localization (Fig 2G). Since the P2A method realizes synchronized translation of CCZ-1 and mCherry, the comparable fluorescence intensity of mCherry indicates that the results are possibly not due to the variations in protein expression level (Fig 2G’). To pinpoint the critical phosphorylation site, we generated glutamic acid substitutions (Tyr-Glu) at each of the eight predicted positions to mimic constitutive phosphorylation. The Y326E mutation notably increased GLO-1 membrane localization, albeit to a slightly lesser extent than high zinc treatment (S2E Fig), suggesting cooperative action among multiple phosphorylation sites or additional mechanisms, such as upregulation of *glo-3* transcription. Taken together, our data suggest that zinc facilitates GLO-1 activation through multiple mechanisms, including the transcriptional upregulation of GLO-3 and the tyrosine phosphorylation of CCZ-1. Notably, because CCZ-1 is also a component of the RAB-7 GEF complex, its regulation may represent a mechanism by which zinc engages RAB-7 to promote its adaptation. This complex regulatory network is expected to ensure efficient GLO-1 activation to fulfill physiological demands in zinc-rich environments.

### Gut granules lacking CDF-2 retain the ability to take up zinc

**Fig 3:**
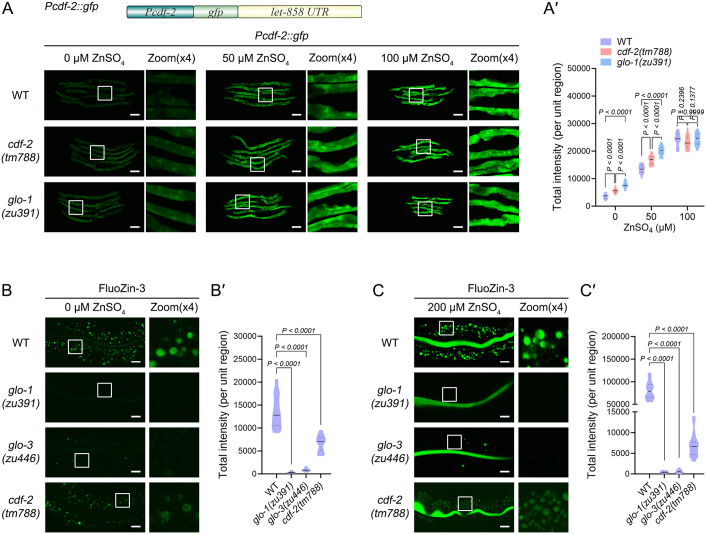
Gut granules retain the ability to take up zinc in the absence of CDF-2. (A-A’) Confocal images showing the fluorescent intensity of the cytosolic zinc reporter *Pcdf-2::gfp*. Statistical analysis of *Pcdf-2::gfp* intensity was shown as mean ± SD (n = 24 circular regions of 35.7 μm diameter from 6 animals; Two-way ANOVA with Bonferroni post-test). Scale bars: 100 μm. (B-C’) Confocal images showing the FluoZin-3 fluorescence intensity. Statistical analysis of FluoZin-3 intensity was shown as mean ± SD (n = 24 circular regions of 11.9 μm diameter from 6 animals; One-way ANOVA test with Dunn’s Multiple Comparison). The confocal microscopy parameters for FluoZin-3: (B) laser power 30%, high voltage 85; (C) laser power 10%; high voltage 70; bit depth was set to 12-bit. Scale bars: 10 μm.

Gut granules rely on the CDF family member CDF-2 for cytosolic zinc import [43, 25]. Loss-of-function mutations in key regulators of gut granule biogenesis, such as *glo-1* and *glo-3*, lead to gut granule loss [30]. Interestingly, *glo-1* mutants exhibit higher zinc sensitivity compared to *cdf-2* mutants [25, 26], prompting further investigation. Thus, we assessed the impact of glo-1 and *cdf-2* mutations on cytosolic zinc levels. The *Pcdf-2::gfp* transcriptional reporter is a well-established indicator of cytosolic zinc concentration [43, 25]. At 0 and 50 μM dietary zinc, *cdf-2* mutants displayed increased GFP fluorescence compared to wild-type animals, while *glo-1* mutants showed even higher fluorescence intensity (Fig 3A). This distinction was not evident at 100 μM zinc, possibly due to limited transgene expression within intestinal cells. Generally, these results suggest that the cytosolic zinc level is more significantly elevated with GLO-1 depletion than with CDF-2 loss.

To assess zinc content within gut granules, we used the labile zinc-sensitive dye Fluozin-3, which predominantly labeled gut granules in *C. elegans* [44]. It was challenging to obtain satisfactory images under consistent microscopy parameters owing to the pronounced difference in Fluozin-3 fluorescence intensity between zinc-deficient and zinc-excess states. Hence, we opted to utilize two sets of parameters for imaging. In the normal diet, wild-type animals showed an obvious Fluozin-3 signal, whereas granule-deficient *glo-1* and *glo-3* mutants showed minimal signal. Notably, *cdf-2* mutants displayed a significantly reduced but still observable Fluozin-3 signal (Fig 3B). This trend persisted under high zinc conditions (200 μM Zinc; Fig 3C). Together, these results affirm that the absence of gut granules has a more pronounced effect on cytosolic zinc levels compared to the *cdf-2* mutants. This indicates that despite the lack of CDF-2, gut granules still possess the ability to uptake zinc, proposing alternative mechanisms for zinc transport into gut granules.

### Evolutionary patterns and intracellular distribution of *C. elegans* CDFs

In light of the supplementary pathways for zinc transport into gut granules apart from CDF-2, it is postulated that other members of the CDF family could potentially be involved in zinc sequestration within gut granules. The *C. elegans* genome encodes 14 CDFs; hence, intestinal-specific GFP-tagged transgenes of these CDFs were generated for a comprehensive examination of their subcellular distributions. Concurrently, based on published research [22], a phylogenetic analysis was conducted to investigate the evolutionary relationships between these CDFs and their human counterparts using the maximum likelihood method (S3 Fig).

**Fig 4:**
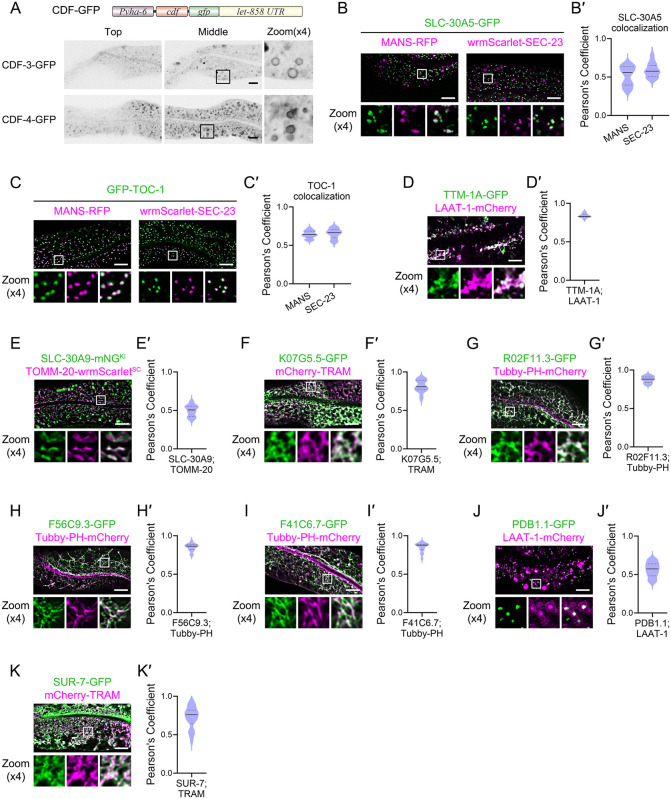
Subcellular distribution of CDFs in *C. elegans* intestinal cells. (A) Confocal images showing the subcellular localization of CDF-3 and CDF-4 within intestinal cells of *C. elegans*. (**B-K’**) Confocal images showing the colocalization of SLC-30A5, TOC-1, TTM-1A, SLC-30A9, K07G5.5, R02F11.3, F56C9.3, F41C6.7, PDB1.1 and SUR-7 with different organelle markers. Pearson’s correlation coefficient was calculated using Fiji software; error bar is 95% CI (n = 18 regions of 3 × 3 μm^2^ from 6 animals). Scale bars: 10 μm.

The results showed that the structures labeled by CDF-3 and CDF-4 are characteristic ring-like structures, which are very likely localized on gut granules (Fig 4A). The localization of other CDFs, except for the reported CDF-1 and CDF-2, lacked significant features. We used colocalization analysis to provide a systematic reference for the subcellular localization of CDF family proteins. Human ZnT5, ZnT6, and ZnT7 localized to the Golgi apparatus [45, 46, 47]. Likewise, the *C. elegans* homologs SLC-30A5 and TOC-1 exhibited *cis*-Golgi and ER exit sites localization (Fig 4B-C). Previous findings reported basolateral membrane localization of CDF-1 [48, 22], which is similar to the plasma membrane targeting of its human homolog ZnT1 [49]. CDF-2 and TTM-1 clustered with human ZnT2/3/4/8 (S3 Fig). ZnT8 was found in secretory vesicles in pancreatic β-cells [50], while ZnT2/3/4 targeted lysosomes [45, 51]. CDF-2 was reported to localize in the lysosome-related organelle, gut granules. Furthermore, colocalization analysis of TTM-1A indicated a lysosome localization (Fig 4D) [22, 52].

The mitochondrial zinc transporter ZnT9 corresponds to *C. elegans* SLC-30A9 [53], which was also localized to mitochondria within intestinal cells (Figs 4E and S3) as in epidermal [24]. Other related CDFs (K07G5.5, R02F11.3, F56C9.3, PDB1.1, F41C6.7) show diverse distributions: K07G5.5 localized to ER (Fig 4F); R02F11.3, F56C9.3, and F41C6.7 localized on basolateral membranes and tubular networks (Fig 4G-I); PDB1.1 localized inside lysosomes, indicating degradation of unknown reason (Fig 4J). In addition, the phylogenetic tree showed relatively independent evolution of SUR-7, which localized on the ER (Fig 4K). Notably, the ring-like patterns exhibited by CDF-3/ZK185.5 and CDF-4/F19C6.5, reminiscent of CDF-2 (Fig 4A), intrigued us to explore their potential functions in zinc transport further.

### CDF-3 and CDF-4 are located in gut granules within intestinal cells

**Fig 5:**
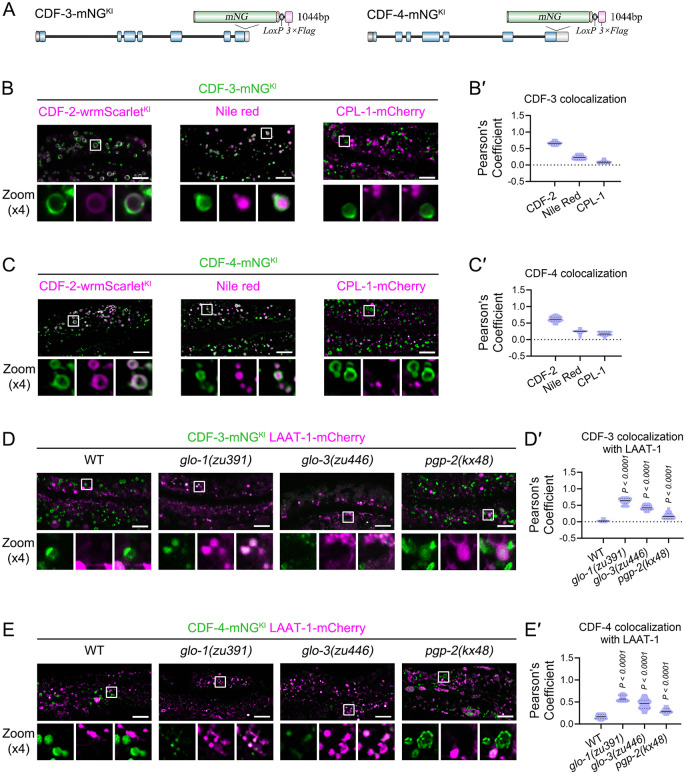
CDF-3 and CDF-4 are situated in gut granules within intestinal cells. (A) Cartoon pictures showing the genomic structure of CDF-3-mNG^KI^ and CDF-4-mNG^KI^. (B-C’) Confocal images showing the colocalization of CDF-3-mNG^KI^ or CDF-4-mNG^KI^ with CDF-2-wrmScarlet^KI^, Nile Red, and CPL-1-mCherry. Pearson’s correlation coefficient was calculated; error bar is 95% CI (n = 18 regions of 3 × 3 μm^2^ from 6 animals). (D-E’) Confocal images showing the colocalization of CDF-3-mNG^KI^ or CDF-4-mNG^KI^ with LAAT-1-mCherry in different genetic backgrounds. Pearson’s correlation coefficient was calculated using Fiji software; error bar is 95% CI (n = 18 regions of 3 × 3 μm^2^ from 6 animals, One-way ANOVA test with Dunn’s Multiple Comparison). The confocal microscopy parameters: CDF-3-mNG^KI^, laser power 25%, high voltage 85; CDF-4-mNG^KI^, laser power 30%, high voltage 95; CDF-2-wrmScarlet^KI^, laser power 30%, high voltage 90; Nile Red, laser power 10%, high voltage 70; LAAT-1-mCherry, laser power 20%, high voltage 80; CPL-1-mCherry, laser power 25%; high voltage 85; bit depth was set to 12-bit. Scale bars: 10 μm.

To determine the expression profile and subcellular localization of CDF-3 and CDF-4, we generated endogenously tagged strains (CDF-3-mNG^KI^ and CDF-4-mNG^KI^) by CRISPR-Cas9-mediated knock-in of mNG-3xFlag before the stop codons [54] (Fig 5A). Imaging analysis revealed predominant intestinal expression of both CDF-3 and CDF-4, with no detectable fluorescence in other examined tissues, including the pharynx, hypodermis, gonad, vulva, or anus (S4A-B Fig). Colocalization studies showed a significant overlap between CDF-3-mNG^KI^ and CDF-2-wrmScarlet^KI^, with CDF-3 forming ring-like structures surrounding the gut granule marker Nile Red (Fig 5B). The lysosomal transporter LAAT-1 and luminal protease CPL-1 (cathepsin L) were used as reference markers for lysosomal compartments. In contrast, CDF-3-mNG^KI^ displayed no significant colocalization with LAAT-1 or CPL-1 (Fig 5B and D). Additionally, our study showed that the subcellular localization of CDF-4 resembled that of CDF-3 (Fig 5B-E). Consistent with these findings, CDF-3 and CDF-4 displayed disrupted patterns in gut granule-loss *glo-1*, *glo-3, pgp-2* mutants [55, 40] (S4C-D Fig). To enhance the comprehension of morphological alterations, colocalization analysis was employed to assess the level of overlap between CDF-3 or CDF-4 and the lysosomal marker LAAT-1-mCherry. The analysis revealed significant translocation of CDF-3 and CDF-4 to lysosomes in *glo-1* and *glo-3* mutants, with a milder effect in the *pgp-2* mutants (Fig 5D-E). These findings imply a distinct involvement of PGP-2 in gut granule biogenesis compared to GLO-1 and GLO-3. Additionally, if unable to reach granules, the transmembrane proteins CDF-3 and CDF-4 are likely directed to the lysosome. These findings further substantiate the gut granule localization of CDF-3 and CDF-4.

In the trans-Golgi network, the AP-3 adaptor recognizes cytoplasmic sorting motifs in LRO-destined proteins [30]. Sequence analysis of CDF-3 identified four putative AP-3 interaction motifs (S4E Fig) [56]. To determine the motif responsible for CDF-3’s localization to gut granules, we generated four mutation forms (LLPLI6AAPAA, Y40A, LL56AA, L317A) (S4F Fig). Our findings revealed that the Y40A and LL56AA mutations had no significant impact on CDF-3 localization, whereas the L317A mutation disrupted vacuolar localization, leading to a predominance of puncta structures (S4G Fig). Notably, the LLPLI6AAPAA mutation abolished ring-like structures, causing a shift to a reticular network pattern (S4G Fig). These data suggest that these two [DE]XXXL[LI] motifs are crucial for mediating the gut granule targeting of CDF-3. Taken together, these results confirm the specific localization of CDF-3 and CDF-4 to gut granules in intestinal cells.

### CDF-3 exhibits the capacity for cytosolic zinc influx into gut granules

**Fig 6:**
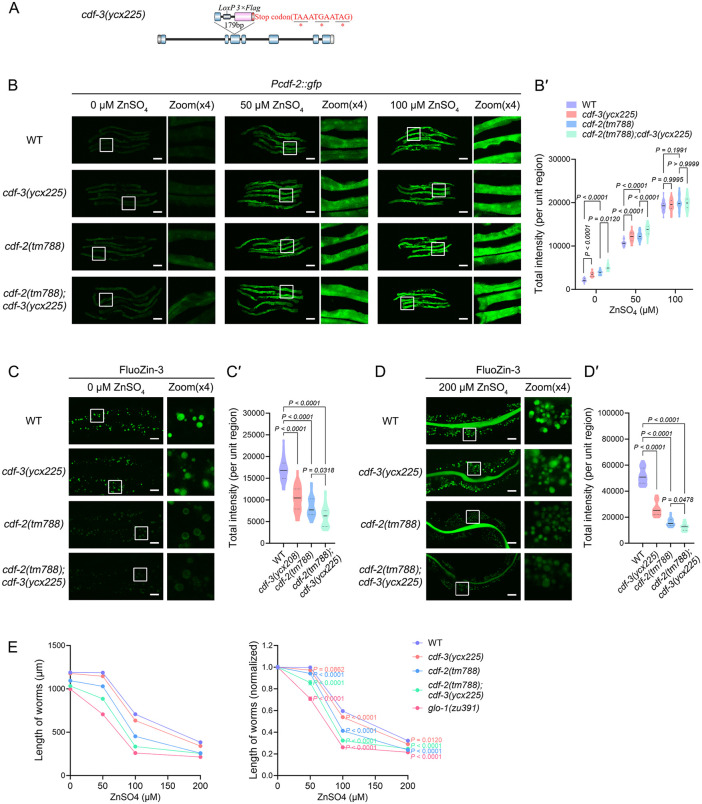
CDF-3 shows the capability for cytosolic zinc influx into gut granules. (A) Cartoon picture showing the genomic structure of *C. elegans cdf-3* and molecular details of *cdf-3(ycx225)*. (B-B’) Confocal images showing the fluorescent intensity of *Pcdf-2::gfp* in different genetic backgrounds with different zinc concentrations. Statistical analysis was shown as mean ± SD (n = 24 circular regions of 35.7 μm diameter from 6 animals; Two-way ANOVA with Bonferroni post-test). Scale bars: 100 μm. (C-D’) Confocal images showing the intracellular FluoZin-3 fluorescence intensity. Statistical analysis of FluoZin-3 intensity was shown as mean ± SD (n = 24 circular regions of 11.9 μm diameter from 6 animals; One-way ANOVA test with Dunn’s Multiple Comparison). The confocal microscopy parameters for FluoZin-3: (C) laser power 30%, high voltage 85; (D) laser power 10%, high voltage 70; bit depth was set to 12-bit. Scale bars: 10 μm. (E-E’) The actual and normalized Length of individual worms of different genetic backgrounds with different zinc concentrations was measured. Statistical analysis was shown as mean ± SD (3 independent biological groups, n = 50; Two-way ANOVA with Bonferroni post-test). Scale bars: 10 μm.

To explore the physiological functions of CDF-3 and CDF-4, we generated *cdf-3* and *cdf-4* null mutants using CRISPR-Cas9 genome editing (Figs 6A and S5B). The zinc-responsive *Pcdf-2::gfp* reporter was then utilized to evaluate the influence of these mutants on cytosolic zinc levels (Fig 6B). Remarkably, depletion of CDF-3 led to a notable increase in *Pcdf-2::gfp* fluorescence intensity compared to the wild-type counterparts under 0 and 50 μM zinc conditions (Fig 6B), indicating heightened cytosolic zinc levels. Moreover, the simultaneous loss of CDF-2 and CDF-3 exhibited a certain degree of phenotypic additive effect (Fig 6B). Given its distribution in the gut granule membrane, we postulated that CDF-3, like CDF-2, facilitates the transport of cytosolic zinc into the gut granule lumen. It is worth noting that the impact of *cdf-3* mutants was less pronounced than that of *cdf-2* mutants at 0 μM zinc, implying that CDF-3 is less potent than CDF-2 under standard culture conditions. Then, we opted to measure gut granule luminal zinc in wild-type, *cdf-3*, *cdf-2*, and double mutant animals using the zinc-sensitive dye Fluozin-3. In both low and high zinc conditions, the Fluozin-3 signal was significantly reduced in *cdf-3* mutants, despite a notably higher zinc level than in *cdf-2* mutants (Fig 6C-D). It is noteworthy that an additive effect was also observed in *cdf-2;cdf-3* double mutants, indicating their parallel operation (Fig 6C-D). These findings collectively support the notion that CDF-3 facilitates the transport of zinc into gut granules, albeit with slightly lower efficacy than CDF-2. Excess environmental zinc has been shown to induce growth retardation [25]. Consistently, knockout of CDF-3, similar to *cdf-2* mutants, heightened zinc sensitivity (Fig 6E). Loss of CDF-3 in a *cdf-2* mutant background further increased sensitivity, which approached the effect of *glo-1* mutant (Fig 6E). Furthermore, the *cdf-3* mutants did not exhibit altered sensitivity to other metal ions compared to wild-type worms, indicating the specificity of CDF-3 in zinc transportation (S5B Fig). Together, these results establish CDF-3 as a new zinc transporter in gut granules that, along with CDF-2, facilitates zinc storage in *C. elegans*.

Regarding the role of CDF-4 in zinc transport, we also assessed the levels of zinc in the cytosol and gut granules following CDF-4 depletion (S5B Fig). However, *cdf-4* mutants failed to display significant differences in *Pcdf-2::gfp* or Fluozin-3 intensity compared to the control group (S5C-E Fig). Likewise, in the zinc detoxification test, the loss of CDF-4 did not impact the zinc sensitivity of the animals (S5F Fig). These findings suggest that although CDF-4 is present in gut granules, it does not play a significant role in facilitating the transport of cytosolic zinc into gut granules, in contrast to CDF-2 and CDF-3. The functional divergence of CDF-4 is in line with its specific evolutionary course, as indicated by phylogenetic analysis demonstrating a notable deviation from other CDFs (S3 Fig).

### The expression of CDF-3 is suppressed by zinc exposure

**Fig 7:**
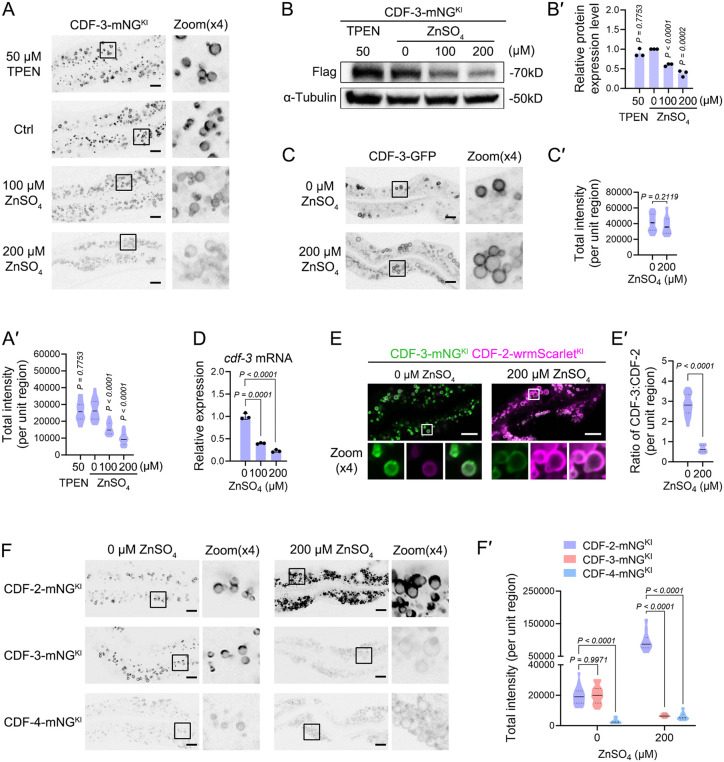
Zinc exposure suppresses the expression of CDF-3. (A-A’) Confocal images showing the subcellular distribution of CDF-3-mNG^KI^ after treatment with different concentrations of zinc. Statistical analysis was shown as mean ± SD (n = 24 circular regions of 11.9 μm diameter from 6 animals; One-way ANOVA test with Dunn’s Multiple Comparison). (B-B’) Western blot analysis of CDF-3 protein levels with different concentrations of zinc using an anti-Flag antibody. The band intensity was measured using the “Plot Lanes” function of Fiji from three independent experiments. The error bars represent 95% CIs (two-tailed unpaired Student’s *t*-test). (C-C’) Confocal images showing the subcellular distribution of CDF-3-GFP after treatment with different concentrations of zinc. Statistical analysis was shown as mean ± SD (n = 24 circular regions of 11.9 μm diameter from 6 animals; two-tailed Mann-Whitney test). (D) Quantitative PCR showing *cdf-3* mRNA expression levels of the Bristol N2 strain. The error bars represent 95% CIs (two-tailed unpaired Student’s *t*-test). (E-E’) Confocal images showing colocalization between CDF-3-mNG^KI^ and CDF-2-wrmScarlet^KI^ with different zinc concentrations. Statistical analysis of the ratio of CDF-3:CDF-2 was calculated using Fiji software and shown as mean ± SD (n = 24 regions of 3 × 3 μm^2^ from 6 animals; two-tailed Mann-Whitney test). (F-F’) Confocal images showing the intensity of CDF-2-mNG^KI^, CDF-3-mNG^KI^ and CDF-4-mNG^KI^ with different zinc concentrations. Statistical analysis was shown as mean ± SD (n = 24 circular regions of 11.9 μm diameter from 6 animals; Two-way ANOVA with Bonferroni post-test). Scale bars: 10 μm.

Organisms regulate zinc transporter expression in response to environmental zinc level changes. In *C. elegans*, increased zinc levels activate the transcription factor HIZR-1, leading to the upregulation of CDF-2 to decrease cytoplasmic zinc concentrations [27, 28]. Therefore, we were interested in exploring how the expression of CDF-3 is influenced by zinc exposure. The results demonstrated a concentration-dependent decrease in CDF-3-mNG^KI^ fluorescence upon zinc exposure (Fig 7A), with no similar effect observed for other divalent metals (Cu^2+^, Fe^2+^, Mn^2+^, Cd^2+^, Ca^2+^, Mg^2+^, Ni^2+^) (S6A Fig), highlighting specific zinc-mediated regulation. Immunoblotting validated a decline in CDF-3 expression under high zinc conditions (Fig 7B). Conversely, the zinc chelator TPEN showed no influence on the expression of CDF-3-mNG^KI^ detected by both imaging and immunoblotting, indicating the presence of a physiological baseline level for CDF-3 that is unaltered by zinc deficiency (Fig 7A).

The decrease in CDF-3 induced by zinc may result from either transcriptional repression or protein destabilization. To investigate these possibilities, we utilized a CDF-3-GFP transgene driven by a constitutive intestinal *vha-6* promoter. We observed an increase in granule number and size under high zinc conditions without altering total fluorescence intensity (Fig 7C), thus excluding protein stability regulation. Quantitative RT-PCR revealed a reduction in *cdf-3* mRNA levels in a dose-dependent manner (Fig 7D), confirming transcriptional repression as the primary regulatory mechanism. Moreover, unlike other zinc-repressed genes that rely on the transcription mediator complex protein MDT-15 for suppression [29], the persistent downregulation of CDF-3 following *mdt-15* RNAi treatment suggests the engagement of alternative transcriptional pathways (S6B Fig).

To directly compare the impact of zinc on the levels of gut granule CDFs, the endogenous CDF-3 and CDF-2 (CDF-3-mNG^KI^ and CDF-2-wrmScarlet^KI^) were studied under varying zinc treatments. The results confirmed contrasting responses to zinc exposure in the same context: while CDF-2 fluorescence increased, CDF-3 signals decreased (Fig 7E). A more comprehensive analysis was conducted on three CDFs (CDF-2-mNG^KI^, CDF-3-mNG^KI^, and CDF-4-mNG^KI^). The findings revealed similar basal expression levels of CDF-2 and CDF-3. Under high zinc conditions, a significant ~16-fold difference in expression was observed between CDF-2 and CDF-3, supporting the dominant role of CDF-2 in zinc detoxification (Fig 7F). In contrast, the levels of CDF-4 stayed much lower than CDF-2 and CDF-3 and showed a slight upregulation under high zinc conditions (Fig 7F). Taken together, these results suggest that CDF-2 and CDF-3 jointly mediate cytosolic zinc influx into gut granules under standard culture conditions. In response to high zinc stress, CDF-2 exhibits a substantial increase in expression levels, assuming the primary role in resistance to zinc toxicity.

## Discussion

**Fig 8:**
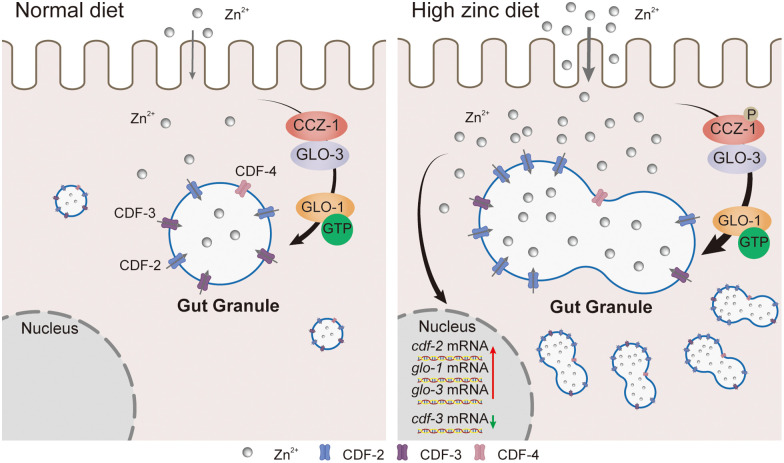
Diverse mechanisms employed by gut granules to uphold cellular zinc homeostasis. Zinc promotes both the transcription and the activity of GLO-1/Rab32/Rab38 to increase gut granule biogenesis, thereby mediating gut granule expansion and population growth under high zinc conditions to provide more space for zinc storage. Further investigation showed zinc modulates GLO-1 activity through its GEF complex by regulating transcription of GLO-3 and phosphorylation of CCZ-1. In addition to CDF-2, other CDF family members, CDF-3 and CDF-4, also localize on the gut granule membranes, with CDF-3 possessing zinc influx functionality. Upon high zinc stimulation, the expression of CDF-2 is upregulated while CDF-3 expression is downregulated, resulting in remodeling of gut granule CDF component, which is adaptive to environmental zinc concentration.

Gut granules act as a pivotal zinc reservoir in *C. elegans*, utilizing various mechanisms to regulate zinc levels. Elevated zinc levels trigger morphological changes in gut granules, suggesting the presence of specialized mechanisms that respond to zinc and potentially enhance their capacity for zinc storage. Our study demonstrates that the intricate zinc response is dependent on GLO-1. Zinc enhances GLO-1 activity in a dose-dependent fashion, resulting in increased gut granule quantity and morphological expansion. Mechanistic analysis reveals that this process necessitates the GEF complex GLO-3-CCZ-1, with zinc playing a dual role in upregulating GLO-3 expression and promoting CCZ-1 tyrosine phosphorylation. CDF-2 is acknowledged as the exclusive importer of zinc within gut granules. Nevertheless, our results indicate that mutants lacking gut granules display heightened sensitivity to zinc compared to *cdf-2* null mutants, implying the presence of supplementary zinc detoxification pathways. Analysis of *C. elegans* CDF members unveiled CDF-3 and CDF-4 as newly discovered gut granule-localized CDFs. Subsequent study showcased a disparity between these paralogs: CDF-3 facilitated zinc transport into granules, whereas CDF-4 did not. In contrast to CDF-2, which can be induced by zinc, CDF-3 showed decreased expression with increasing zinc levels. These findings suggest that CDF-2 predominantly facilitates zinc detoxification under high zinc condition. Therefore, our study demonstrates that gut granules utilize a multifaceted approach to maintain zinc homeostasis, integrating GLO-1 for regulating gut granule quantity and morphology, and CDFs for enhancing zinc influx (Fig 8).

Phosphorylation, a reversible post-translational modification mediated by the opposing actions of kinases and phosphatases, serves as a rapid way to modulate protein functions. Protein-tyrosine phosphatases (PTPs), a critical subclass of phosphatases, are known to be potently inhibited by zinc, as demonstrated for PTP-1B and SHP-1 [57, 58, 59]. This zinc-dependent inhibition of PTP activity leads to concomitant activation of tyrosine kinases, resulting in increased tyrosine phosphorylation of downstream substrates. Here, we observed elevated tyrosine phosphorylation of CCZ-1 under high zinc conditions, a phenomenon that may result from zinc-mediated suppression of PTP activity. However, the specific kinases and phosphatases responsible for regulating CCZ-1 phosphorylation in this context remain to be identified. The zinc-induced modulation of CCZ-1 phosphorylation status represents a plausible mechanism for the rapid activation of GLO-1, thereby enabling gut granules to promptly adapt to fluctuating environmental zinc. The phosphorylation-dependent regulation of GLO-1 activity through CCZ-1 may constitute an important sensory mechanism that allows dynamic and reversible responses to zinc availability, although the complete details of this signaling cascade require further elucidation.

Previous research revealed the subcellular localization of CDF-1, CDF-2, and TTM-1B, with expression patterns and intracellular distribution of the rest of CDFs unknown. We examined additional transcription data [60, 61, 62, 63, 64, 65] sourced from WormBase (version: WS298), which validated that, aside from R02F11.3, all CDFs are likely to be expressed in the intestine. However, Packer JS et al. identified transcription of R02F11.3 in embryonic intestinal cells [66]. To provide comprehensive information for the field, we have supplemented the colocalization analysis data of all CDFs with unclear subcellular localization (Fig 4). It was worth noting that heterologous expression and overexpression can lead to the mis-localization of the CDFs. However, due to the focus of this study, we have only provided data on the distribution of endogenous CDF-3 and CDF-4 (Fig 5). Although the transgene is likely to cause mis-localization, it still holds value as a scientific cue.

Our study demonstrates that CDF-2 is not the sole mediator of zinc sequestration in gut granules. However, the lack of CDF-3 resulted in a less pronounced impact on gut granule zinc accumulation and zinc sensitivity than the absence of CDF-2 (Fig 6), potentially attributed to decreased expression levels in response to a high zinc diet (Fig 7). The divergent expression profiles of CDF-2 and CDF-3 lead to the predominance of CDF-2 under high zinc diet circumstances (Fig 7F). These findings collectively establish CDF-2 as the primary zinc detoxification factor within the gut granules of *C. elegans*. The evolutionary rationale for this strategy may involve selective pressure to maximize zinc transport efficiency within the limited membrane space of gut granules during zinc stress. This may favor increased expression of the potentially more potent CDF-2 while suppressing the less efficient CDF-3. The enhanced sensitivity to zinc in *cdf-2(tm788);cdf-3(ycx225)* worms compared to CDF-2 depletion alone supports the notion that, despite its lower transport capacity, CDF-3 plays a significant role in zinc detoxification. This supplementary function may be particularly important during phases of CDF-2 induction.

The CDF-2-GFP transgene in prior studies showed a polarized distribution on gut granule membranes [25, 39, 26]. Accordingly, we also found the endogenous CDF-2 exhibited a polarized distribution, with a tendency to accumulate predominantly on one side of the granules (Fig 1A). Likewise, endogenous CDF-3 displays asymmetric localization, whereas the restricted expression of CDF-4 hindered the clear determination of its preferred location (Fig 7A and 7F). Conversely, overexpressed CDF-3 shows a more uniform distribution, with fewer bi-lobe-shaped granules labeled by it (Fig 7C). This altered distribution likely results from the overexpression of CDF-3, disrupting its usual polarized localization. Consequently, this modified distribution may cause a uniform influx of zinc ions into granules, resulting in a spherical morphology. Additionally, our observation of partial disruption in polarization upon simultaneous tagging of two endogenous CDFs indicates that fluorescent tags might impede cytoplasmic domains essential for accurate localization (Fig 5B). Of note, several CDF family members function as heterodimers [67]. Although CDF-2 and CDF-3 colocalize on gut granules, it is unclear whether they form dimers and how these interactions are linked to their asymmetric distributions. The clustered subdomains of observed CDFs may indicate specialized functional zones for cytosolic zinc import into gut granules, necessitating further investigation into their biological significance.

Our study identified CDF-4 as a gut granule-localized CDF specifically expressed in intestinal cells. However, genetic mutation of CDF-4 did not significantly affect zinc concentrations in the cytosol or gut granule lumen. Quantitative analysis showed lower protein expression levels of CDF-4 compared to its paralogs (Fig 7F), potentially explaining the lack of observable zinc transport activity. Phylogenetic analysis revealed that CDF-4 is evolutionarily distinct from other CDF family members, suggesting functional specialization. As an integral membrane protein of gut granules, CDF-4 may transport alternative ions or small molecules. These findings highlight the functional diversification of gut granule-localized transporters, with CDF-4 likely possessing a unique substrate specificity separate from zinc transport activity.

## Materials and methods

### General methods and strains

The *C. elegans* strains used in this study were derived from the Bristol N2 strain. Worms were cultured at 20℃ on nematode growth medium (NGM) seeded with *Escherichia coli* OP50 strain as the food source [68]. The following mutations were used in this study: *glo-1(zu391)* [30], *cdf-2(tm788)* [43], *glo-3(zu446)* [40], *pgp-2(kx48)* [55]. *cdf-3(ycx225)* and *cdf-4(ycx137)* were generated using CRISPR/Cas9 technology. The detailed information of the strains was listed in S1 Table.

### Transgenic strains

To achieve the specific expression of the CDF genes in the intestine, cDNA sequences of the target genes were inserted into the *vha-6* promoter-driven destination vectors containing GFP, RFP, or mCherry tag and *let-858 UTR*. The extrachromosomal array transgenic strains were obtained using the standard microinjection technique. *Pcdf-2::gfp* reporter vector was constructed as described [28]. To obtain stable expression of *Pcdf-2::gfp*, low-copy integrated transgenic lines were prepared using the microparticle bombardment method [69].

### CRISPR/Cas9 strains

To generate knock-in (abbreviated as ‘KI’) strains, one or two single-guide RNA (sgRNA) were designed using an online *C. elegans* CRISPR guide RNA selection tool (http://genome.sfu.ca/crispr/search.html) and inserted into the pDD162 vector, which expresses Cas9 nuclease to achieve highly efficient cutting of the target sequence. The sgRNA sequences were listed in S2 Table. For homology recombination-mediated knock-in, 1 kb upstream and downstream homologous arms flanking the cutting site were inserted into the pUC19 vector as the repair template. The coding sequence for mNeonGreen (abbreviated as ‘mNG’) or wrmScarlet with a Self-Excising Drug Selection Cassette (SEC) followed by a 3 × Flag tag was inserted between the two homologous arms [54]. These three plasmids were microinjected into the worm gonad, with the pDD162 vector at a concentration of 10 ng/μl and the pUC19 vector at a concentration of 25 ng/μl. Progenies were identified by following the roller and hygromycin resistance phenotypes and singled into new NGM plates. The next generation progenies were confirmed through genotyping and sequencing. The SEC sequences were eliminated through heat shock. GFP-GLO-1 KI (constructed by CRISPR-cas9, sgRNA: 5’-GATCATAAGCTGAGCTGCTA-3’) was acquired from Dr. Chonglin Yang [34].

To generate gene knock-out mutant strains, two sgRNA sequences (S2 Table) targeting the third exon of the *cdf-3* gene or the second exon of the *cdf-4* gene were cloned into the pDD162 vector, respectively. A repair template was designed to terminate the translation early by adding a stop codon cassette (5’-TAAATGAATAG-3’) [70] after the SEC sequences. The subsequent construction steps of the mutant strains followed the same procedure as described for the knock-in strain.

The pOG2034 vector was constructed by replacing the *eft-3* promoter of the pDD162 vector with the *hsp-16.2* promoter, which could realize heat-shock-induced Cas9 expression. To achieve conditional knockout of genes, three sgRNA sequences targeting exons of *rab-7* (S2 Table) were cloned into the pOG2034 vector. A transgenic animal (ycx229) was generated through the microinjection of these three plasmids at a concentration of 50 ng/μl and a selection marker *Podr-1::gfp* into the gonad of N2 hermaphrodites. Conditional knockout was conducted by heat shocking of *C. elegans* eggs at 32℃ for 1 hour.

The single-copy knock-in strains (abbreviated as ‘SC’) were also constructed using CRISPR-Cas9. The *ttTi4348* genome loci was used as insertion site on chromosomes Ⅰ, and the *ttTi5605* genome loci was used as insertion site on chromosomes Ⅱ [33].

### Zinc and TPEN treatment of worms

Young adult hermaphrodites were collected and washed with M9 buffer, and then treated with NaOH and bleach. The eggs were incubated in M9 buffer overnight to allow hatching and synchronized arrest at the L1 stage. L1 worms were transferred to NGM plates. L4 stage hermaphrodites were then transferred to NAMM plates [71] supplemented with zinc sulfate (ZnSO4), or TPEN (N, N, N’, N’-Tetrakis(2-pyridylmethyl) ethylenediamine, Sigma-Aldrich) and fed with concentrated OP50. After 24 h, the animals were used for further analysis.

### Staining with FluoZin-3 and Nile Red

FluoZin-3 acetoxymethyl (AM) ester (Molecular Probes, Thermofisher) was dissolved in dimethylsulfoxide (DMSO) to generate a 1 mM stock solution that was kept at -30℃, diluted in M9, and dispensed on NAMM dishes to yield a final concentration of 3 μM. The synchronized L4 stage hermaphrodites were cultured on these plates in the dark. After 24 h, the worms were transferred to NAMM plates without FluoZin-3 for 30 min to reduce intestinal luminal retention and then analyzed by fluorescence microscopy.

Nile Red (Molecular Probes, Sigma-Aldrich) was dissolved in acetone to generate a 0.5 mg/ml stock solution that was kept at room temperature, diluted in M9, and dispensed on NGM dishes to yield a final concentration of 0.05 μg/mL. The synchronized L4 stage hermaphrodites were cultured on these plates in the dark. After 24 h, the worms were transferred to NGM plates without Nile Red for 30 min to reduce intestinal luminal retention of Nile Red and then analyzed by fluorescence microscopy.

### Confocal microscopy and image analysis

For live fluorescence microscopy, live worms were placed on a 2% agarose pad containing a drop of 20 mM levamisole and imaged with a Nikon C2 laser scanning confocal microscope equipped with a 10× (NA 0.3) objective and a 100× (NA 1.4) oil objective. Z-stack optical sections were captured at 1.0 μm intervals with excitation wavelengths of 405, 488, 561, or 594 nm. Images were captured using NIS-Elements Advanced Research version 4.40.00 software (Nikon). We utilized narrow-spectrum emission detection - specifically 525 ± 10 nm for green channels and 605 ± 10 nm for red channels - which selectively captures the peak emission of reporters while minimizing the broader autofluorescent tail. To track the dynamics of gut granules, live worms were imaged with a spinning-disk confocal microscope (Olympus IXplore SpinSR) equipped with a 100× (NA 1.45) oil objective. Time series were recorded using Olympus cellSens Dimension software. All images were taken at room temperature. Unless otherwise mentioned, Metamorph version 7.8.0.0 software (Universal Imaging) was used to analyze fluorescence intensity (total intensity) within unit regions. A uniform color threshold to just cover the target structure was chosen for different groups in each experiment. A total of 24 random regions from six animals per genotype were collected for analysis. Fiji (ImageJ) software was used to analyze the colocalization using line profiling across the region of interest, or the JACoP plugin to calculate Pearson’s coefficient.

### Quantitative real-time PCR (RT-qPCR)

Total RNAs were extracted using the RNA Easy Fast Tissue/Cell Kit (TIANGEN). cDNAs were synthesized using the PrimeScript FAST RT reagent Kit with gDNA Eraser (TAKARA). Real-time qPCRs were performed using TB Green Premix Ex Taq (Tli RNaseH Plus) (TAKARA) and monitored with the CFX Connect Real-Time PCR Detection System (Bio-Rad). mRNA levels of target genes were normalized by the *act-5* mRNA. qPCR was performed with the following primers: *act-5*, 5’-CAACATTCAGGCTGTGCTTT-3’ and 3’-TGATGGATTGGTAGGTGGTCT-5’; *glo-1*, 5’-CAAGGGAACGCACTAT-3’ and 3-CCTCCTCAATGCCAAC-5’; *glo-3*, 5’-ATGGACGACAAGACGA-3’ and 5’-GAGGGAGTGTAGATAGTTT-3’; *ccz-1*, 5’-CCTGCTCTAATGTGGTC-3’ and 5’-TGTTATTTCGGTCTGTCT-3’; *cdf-3*, 5’-AGGAATCACGCAACCG-3’ and 5’-GACAGCCGATCCACCA-3.’

### *C. elegans* western blotting

Synchronized CDF-3-mNG-3xFlag adult worms were collected and washed with M9 buffer. The worm pellet was resuspended in 5 volumes of ice-cold RIPA lysis Buffer (Beyotime) containing 1 mM PMSF and protease inhibitor cocktail (Sigma-Aldrich). The pellet was lysed using an automatic grinding machine (JX-FSTPRP; Jingxin Inc.) at 65 Hz for 15 min (10 s interval after every minute). The lysate was incubated on ice for 10 min and then centrifuged at 12,000 × g for 10 min. A protein loading buffer was added to the supernatant. After boiling at 100 ℃ for 10 min, the samples were analyzed using SDS-PAGE (10% [w/v] polyacrylamide) and blotted onto PVDF membranes. The immunoblot was probed with mouse anti-Flag monoclonal antibody (F1804; Sigma-Aldrich) and mouse anti-α-tubulin monoclonal antibody (T6199; Sigma-Aldrich).

### Phosphorylation assay

Synchronized CCZ-1-GFP and GLO-3-GFP transgene adult worms were collected and washed with M9 buffer. The pellet was resuspended in 5 volumes of ice-cold RIPA lysis Buffer (Beyotime) containing 1 mM PMSF, protease inhibitor cocktail (Sigma-Aldrich), and phosphatase inhibitor cocktail (Biosharp). The pellet was lysed as described. After centrifugation, the protein lysate was incubated with 25 μl of GFP-Nanoab-agarose beads (LabLead) at 4℃ overnight. The precipitates were washed five times with lysis buffer and were subjected to SDS-PAGE and western blotting analysis. The blot was probed with mouse anti-phospho-tyrosine monoclonal antibody (PY99) (sc-7020; Santa Cruz), mouse anti-phospho-threonine monoclonal antibody (H-2) (sc-5267, Santa Cruz), mouse anti-phospho-serine monoclonal antibody (16B4) (sc-81514, Santa Cruz), and mouse anti-GFP monoclonal antibody (AG281; Beyotime).

### RNA interference (RNAi)

RNA interference (RNAi) was induced through feeding the *C. elegans* strains with *E. coli* HT115 carrying the L4440 vector that expresses the double-stranded RNA (dsRNA) targeting the gene of interest [72]. The inserting DNA sequences for targeting of *glo-1*, *glo-3*, *ccz-1*, *ccz-1 3’UTR*, *rab-7*, or *mdt-15* were listed in S2 Table. HT115 carrying the empty L4440 vector was used as the negative control. HT115 carrying L4440 vectors were cultured on NGM plates supplemented with IPTG to induce dsRNA expression. 3–5 young adult hermaphrodites were placed on plates seeded with HT115, and the F1 offspring were used for further analysis.

### Zinc sensitivity assay

Zinc sensitivity of worms was detected as described [25]. Synchronized L1 worms were transferred to NAMM plates supplemented with zinc sulfate (ZnSO_4_) and fed with concentrated OP50. After 72 hours, brightfield images of the worms were acquired using Nomarski microscopy. Length of individual worms was measured as an indicator of growth using Fiji (ImageJ) software by drawing a line from the nose to the tail tip.

### Metal treatment of worms

To analyze the effects of copper (Cu^2+^) on *C. elegans*, synchronized L4 hermaphrodites were transferred to NGM plates supplemented with 500 μM or 2 mM CuSO_4_ [73] and fed with inactivated OP50 (70℃ for 4 h). After 24 h, the animals were used for imaging. As for studying the effects of ferrous (Fe^2+^) and cadmium (Cd^2+^) ions, synchronized L4 hermaphrodites were transferred to NGM plates supplemented with 100 μM or 200 μM FeCl_2_, or 30 μM CdCl_2_ [74] and fed with concentrated OP50. After 24 h, the animals were able to be used for further analysis. To detect the effects of calcium (Ca^2+^) and magnesium (Mg^2+^), eggs were transferred to NGM plates supplemented with 5 mM or 10 mM CaCl_2_) or 5 mM or 40 mM MgSO_4_ and fed with concentrated OP50. The worms were used for further analysis 72 h later [75]. For nickel (Ni^2+^) and manganese (Mn^2+^), synchronized young adult hermaphrodites were treated with an 85 mM NaCl solution containing 2.5 mM or 5 mM NiCl_2_, or 5 mM or 10 mM MnCl_2_, on a rotator at 20℃. After 1 h, worms were washed with 85 mM NaCl and transferred to new NGM plates for 2 h for imaging [76–82].

To assess the metal ion transport specificity, wild type and *cdf-3(ycx225)* mutant worms were treated with different ions as described above. Synchronized L1 worms were treated with different ions. 48 h after treatment, the length of individual worms was measured as an indicator of growth using Fiji software.

### Statistical analysis

GraphPad Prism 8.0.2 software was used for statistical analysis. Biochemical datasets were assessed using a Student’s *t*-test. For imaging datasets with only two variables, a Mann-Whitney U test was used. Alternatively, One-way ANOVA followed by a Dunn’s post hoc multiple comparison test was conducted for imaging datasets with more than two variables. In addition, a Two-way ANOVA test was employed for grouped datasets.

## Supporting information

S1 Fig Membrane trafficking contributes to gut granules’ adaptation to high zinc condition. (A) Spinning-disk confocal images showing the dynamics of CDF-2 and Nile Red. Arrowheads represent the movement of CDF-2-labeled gut granule. (B) Confocal images showing the subcellular distribution of CDF-2-mNG^KI^ and Nile Red. Statistical analysis was shown as mean ± SD (n = 24 regions of 3 × 3 μm^2^ from 6 animals; Two-way ANOVA with Bonferroni post-test). Scale bars: 10 μm.
(TIF)

S2 Fig High dietary zinc activates GLO-1 in a GEF-dependent manner. (A) Confocal images showing the fluorescent intensity of *Pvha-6::gfp* with different zinc concentrations. Statistical analysis of *Pvha-6::gfp* intensity was shown as mean ± SD (n = 24 circular regions of 11.9 μm diameter from 6 animals; One-way ANOVA test with Dunn’s Multiple Comparison). (B-C’) Confocal images showing the subcellular distribution of GLO-1-GFP or GFP-GLO-1 in different genetic backgrounds. Statistical analysis was shown as mean ± SD (n = 24 circular regions of 11.9 μm diameter from 6 animals; One-way ANOVA test with Dunn’s Multiple Comparison). (D) Immuno-precipitation of GLO-3-GFP and western blot analysis of its phosphorylation state. (E) Confocal images showing the intensity of GFP-GLO-1^SC^ with different glutamic acid substitution mutants. Statistical analysis was shown as mean ± SD (n = 24 circular regions of 11.9 μm diameter from 6 animals; One-way ANOVA test with Dunn’s Multiple Comparison). Scale bars: 10 μm.
(TIF)

S3 Fig Evolutionary patterns and subcellular positioning of *C. elegans* CDFs. The phylogenetic tree illustrates the evolutionary relationships among CDFs in *H. sapiens* and *C. elegans* using the maximum likelihood method in MEGA 12.0.11 software. The numbers above the branches represent bootstrap values (expressed as percentages) based on 1000 replicates. The scale bar represents genetic distance.
(TIF)

S4 Fig Expression pattern of CDF-3-mNG^KI^ and CDF-4-mNG^KI^ across tissues. (A-B) Confocal images showing the expression of CDF-3-mNG^KI^ or CDF-4-mNG^KI^ in the *C. elegans* pharynx, intestine, hypodermis, gonad, vulva, and anus. Line scan profiles of the white line were conducted using Fiji software. (C-D’) Confocal images showing the subcellular distribution of CDF-3-mNG^KI^ or CDF-4-mNG^KI^ in different genetic backgrounds. Statistical analysis of the diameter of CDF-3 or CDF-4 structures was calculated manually and shown as mean ± SD (n = 24 regions of 3 × 3 μm^2^ from 6 animals; One-way ANOVA test with Dunn’s Multiple Comparison). (E) Protein sequence and motifs of CDF-3. (F) Cartoon picture showing the protein structure of CDF-3 with the potential motifs recognized by adaptor proteins. (G) Confocal images showing the distribution of CDF-3 with different alanine substitution mutants. Scale bars: 10 μm.
(TIF)

S5 Fig CDF-4 does not mediate the cytoplasmic zinc translocation into the gut granule. (A) Length of individual worms in different genetic backgrounds with different zinc concentrations was measured using Fiji software. To compare the growth rate of different strains with different zinc concentrations, we normalized the length by setting the value at 0 μM Zinc equal to 1.0 for each strain. The statistical analysis of the length of worms after treatment with distinct divalent cations was shown as mean ± SD (n = 20 animals; Two-way ANOVA with Bonferroni post-test). (B) Genomic structure of *C. elegans cdf-4* and molecular details of *cdf-4(ycx137)*. (C-C’) Confocal images showing the fluorescent intensity of the cytosolic zinc reporter *Pcdf-2::gfp* in different genetic backgrounds with different zinc concentrations. Statistical analysis of *Pcdf-2::gfp* intensity was shown as mean ± SD (n = 24 circular regions of 35.7 μm diameter from 6 animals; Two-way ANOVA with Bonferroni post-test). Scale bars: 100 μm. (D-E’) Confocal images showing the FluoZin-3 fluorescence intensity. Statistical analysis was shown as mean ± SD (n = 24 circular regions of 11.9 μm diameter from 6 animals; One-way ANOVA test with Dunn’s Multiple Comparison). Scale bars: 10 μm. (F-F’) Confocal images showing the length in different genetic backgrounds with different zinc concentrations. Length of individual worms was measured using Fiji software. To compare the growth rate of different strains with different zinc concentrations, we normalized the length by setting the value at 0 μM Zinc equal to 1.0 for each strain. Statistical analysis was shown as mean ± SD (n = 20 animals). Scale bars: 100 μm.
(TIF)

S6 Fig CDF-3 expression is insensitive to non-zinc divalent cations and independent of the transcription factor MDT-15. (A-A’) Confocal images showing the subcellular distribution of CDF-3-mNG^KI^ after treatment with distinct divalent cations. Statistical analysis was shown as mean ± SD (n = 24 circular regions of 11.9 μm diameter from 6 animals; One-way ANOVA test with Dunn’s Multiple Comparison). (B- B’) Confocal images showing the intensity of CDF-3-mNG^KI^ after RNAi-mediated knockdown of *mdt-15*. Statistical analysis was shown as mean ± SD (n = 24 circular regions of 11.9 μm diameter from 6 animals; Two-way ANOVA with Bonferroni post-test). Scale bars: 10 μm.
(TIF)

S1 Table Table of strains used in this study. (XLSX)

S2 Table List of DNA sequences. (XLSX)

S3 Table Raw numerical data. (XLSX)

S1 Movie Time-lapse analysis of CDF-2 labeled structure dynamics at 0 μM Zinc. (AVI)

S2 Movie Time-lapse analysis of CDF-2 labeled structure dynamics at 200 μM Zinc. (AVI)

S1 Raw images Raw images of blots. (PDF)
