# Supplementary figures and images for: Lysosome-related organelles employ divergent mechanisms to modulate cytosolic zinc homeostasis
Source: PLoS Genet. 2026 Jun 22;22(6):e1012199. doi: 10.1371/journal.pgen.1012199 (PMC13286216; doi:10.1371/journal.pgen.1012199)

Fig 2E

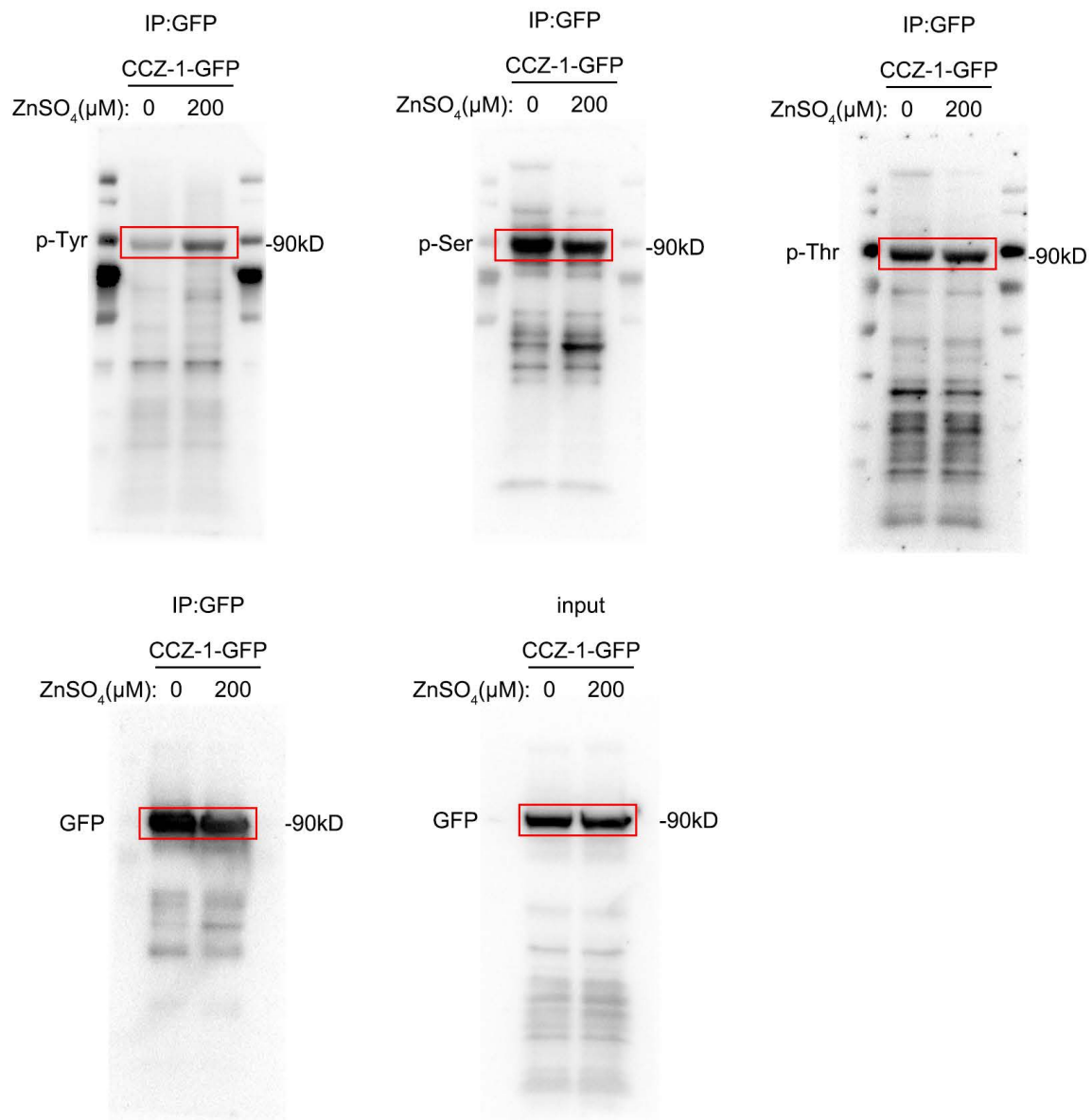

Fig 7B

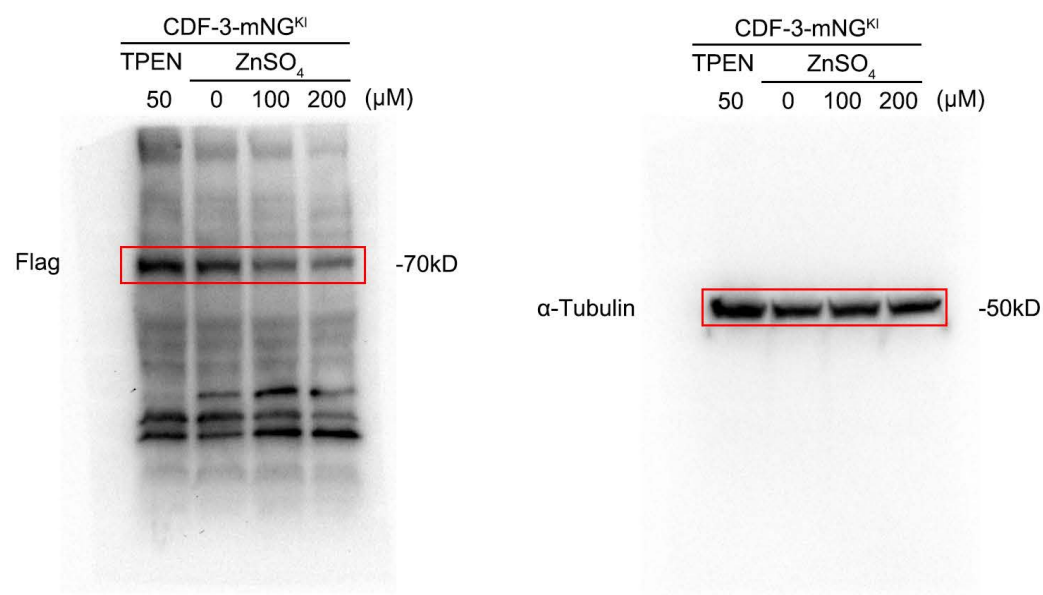

Fig S2D

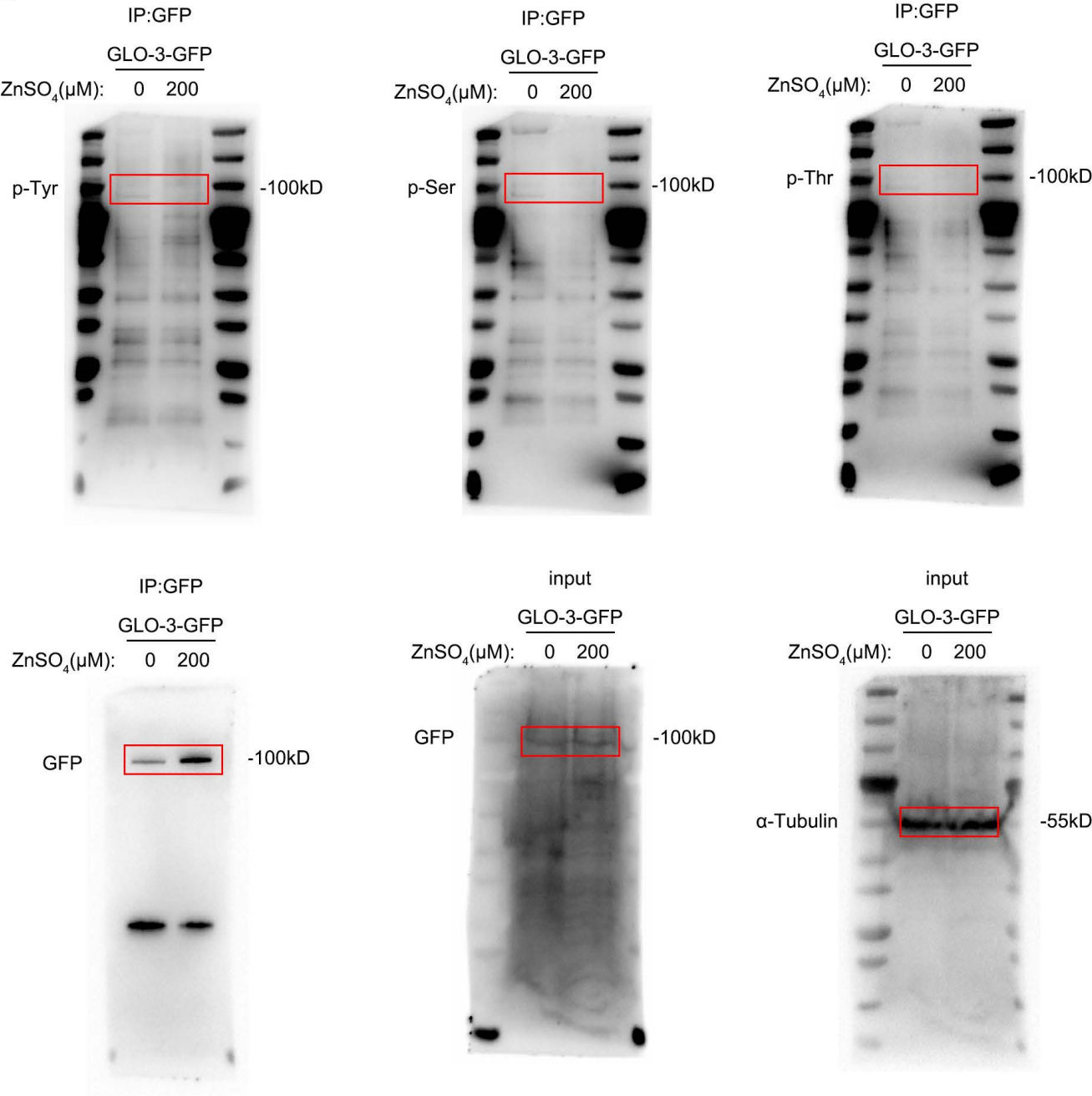

Supplement: S1 Raw images — (PDF) [file pgen.1012199.s012.pdf]
